# Retraction: Response of methane production via propionate oxidation to carboxylated multiwalled carbon nanotubes in paddy soil enrichments

**DOI:** 10.7717/peerj.4267/retraction

**Published:** 2018-01-23

**Authors:** PeerJ Editorial Office

Retraction: Zhang J, Xia X, Li S, Ran W. (2018) Response of methane production via propionate oxidation to carboxylated multiwalled carbon nanotubes in paddy soil enrichments. PeerJ 6:e4267 10.7717/peerj.4267

Immediately after publication, readers alerted the authors that the nanotubes used in their experiments may not have possessed the electrical conductivity that the authors had assumed (based on the manufacturer’s data). The authors tested the nanotubes in question, and confirmed that their electrical conductivity is too low to be able to rely on their results. As a result, the conclusions in the article cannot be relied upon, and all the authors have agreed to retract this article. The authors would like to thank the readers who alerted them to this issue.

This Retraction updates an Expression of Concern which was issued on January 18th 2018.

